# Towards Enhancing Drug Development Methodology to Treat Cognitive Impairment Associated With Schizophrenia and Other Neuropsychiatric Conditions: Insights From 2 Decades of Clinical Trials

**DOI:** 10.1093/schbul/sbae151

**Published:** 2025-02-21

**Authors:** William P Horan, Amir Kalali, Stephen K Brannan, Wayne Drevets, Matthew Leoni, Atul Mahableshwarkar, William J Martin, Srinivas Rao, Corey Reuteman-Fowler, Colin Sauder, Adam Savitz, Jaskaran Singh, Jane Tiller, Gary Walker, Jens R Wendland, Philip D Harvey

**Affiliations:** Karuna Therapeutics, A Bristol Meyers Squibb Company, USA; University of California, Los Angeles, USA; International Society for CNS Drug Development, San Diego, USA; Janssen Research & Development, LLC, a Johnson & Johnson company, San Diego, USA; Janssen Research & Development, LLC, a Johnson & Johnson company, San Diego, USA; Cerevel Therapeutics, Boston, USA; Cybin IRL, Dublin, Ireland; Johnson & Johnson, Inc., San Diego, CA, USA; atai Life Sciences, Berlin, Germany; Boehringer Ingelheim, Ridgefield, USA; Karuna Therapeutics, A Bristol Meyers Squibb Company, USA; Alto Neuroscience, Los Altos, USA; Neurocrine Biosciences, San Diego, CA, USA; Janssen Research & Development, LLC, a Johnson & Johnson company, San Diego, USA; Recognify Life Sciences, San Francisco, USA; Kynexis Therapeutics, Cambridge, USA; University of Miami Miller School of Medicine, Miami, USA

**Keywords:** cognition, imaging, clinical trials, drug development

## Abstract

Cognitive impairment is a core feature and leading cause of functional disability in schizophrenia and other neuropsychiatric disorders. The Measurement and Treatment Research to Improve Cognition in Schizophrenia (MATRICS) initiative in the early 2000s marked a pivotal moment for drug development, establishing consensus on methodology for treatment studies, including assessment strategies and trial designs, for cognitive impairment associated with schizophrenia (CIAS). Despite extensive industry-sponsored and academic drug development efforts over the last 2 decades using these strategies no pharmacological treatments have been approved for CIAS. Drawing on pharmaceutical industry experience and scientific developments since the MATRICS initiative, we review lessons learned about the practical and operational complexities of conducting large-scale CIAS clinical trials. Based on this collective experience, we identify elements of the MATRICS guidelines that may warrant reconsideration and suggest some new approaches to streamline the drug development pathway, without weakening standards for evidence. Our goal is to initiate an open exchange among all stakeholders about possible enhancements to drug development methodology that optimize our ability to develop new treatments for cognitive impairment in schizophrenia and other neuropsychiatric disorders.

## Introduction

Cognitive impairment is a pervasive feature of schizophrenia, significantly impacting patients’ functional outcomes and quality of life.^1,2^ A similar profile of functionally disabling cognitive impairment extends to other neuropsychiatric disorders, particularly bipolar disorder and major depressive disorder (MDD) (even during euthymic states and in unimpaired relatives^3–7^). Despite its well-established functional consequences, pharmacological treatments targeting cognitive impairment associated with schizophrenia (CIAS) and other neuropsychiatric disorders remain elusive^8,9^ as there are no regulatory agency-approved therapies to treat CIAS.

Over the past 2 decades, substantial efforts have been made to develop novel treatments, with the Measurement and Treatment Research to Improve Cognition in Schizophrenia (MATRICS) initiative representing a landmark achievement in this endeavor.^10,11^ Through consensus-building among diverse stakeholders (which included representatives from academia, the National Institutes of Health, and the US Food and Drug Administration [FDA]), MATRICS laid the groundwork for standardized assessment methodologies and clinical trial designs. However, the translation of these efforts into effective treatments has met with numerous challenges, including operational complexities, scientific and conceptual uncertainties, and replication failures in later-stage trials.

In this article, we review lessons learned from conducting industry-sponsored clinical trials for CIAS and other neuropsychiatric disorders, and relevant scientific findings, that have emerged since the MATRICS guidelines were established based on the information available at that time. Based on these new developments, we identify elements of the guidelines that may warrant reconsideration and suggest some new approaches that could help optimize the drug development process, without relaxing standards for evidence. This article is the result of an in-person workshop held during the 22nd annual meeting of the International Society for CNS Drug Development in March 2024. The goal is to initiate open exchange among diverse stakeholders who share a commitment to developing new treatments for CIAS and other neuropsychiatric conditions.

## The MATRICS Initiative: A Milestone in CIAS Treatment Development

The MATRICS initiative emerged in response to the urgent need for standardized assessment tools and clinical trial methodologies for CIAS. Key outcomes of the initiative included^12,13^:

- Identification of 7 cognitive subdomains agreed to be essential for assessment.
- Development of the MATRICS Consensus Cognitive Battery (MCCB) to assess these domains.
- Requirement for a co-primary measure to demonstrate the functional significance of any changes in cognitive performance.
- Guidelines for patient selection, emphasizing the enrollment of clinically stable patients (to address pseudo-specificity concerns) and suggesting the inclusion of participants with all cognitive ability levels (unless impairment is so severe that it compromises the validity of assessment).
- A recommended minimum trial duration of 6 months.
- Clear guidance on the evaluation of adjunctive therapies through multiple double-blind, placebo-controlled augmentation trials of on-going stable antipsychotic treatment.

These standards paved the way for industry-sponsored clinical trials targeting CIAS, catalyzing substantial investments in drug development. Despite considerable subsequent efforts, dozens of adjunctive treatment trials targeting a variety of empirically selected neurobiological targets have been plagued by replication failures, as development programs advanced from small academic and proof of concept studies to larger industry-sponsored multi-site and multi-national trials.^8,9^ Only 2 compounds (encenicline and iclepertin) have reached phase 3 trials^14,15^ and there have been no regulatory approvals for CIAS during this nearly 20-year period. Outside of schizophrenia, 1 molecule (vortioxetine) has text in the label indicating that MDD patients treated with this agent demonstrated improvement on the digit symbol substitution test (DSST), a neuropsychological test that largely measures processing speed.

An analysis of this discouraging history reveals that regulatory CIAS trials are operationally complex, practically challenging for both patients and raters, and expensive to implement. Furthermore, detection of a cognitive treatment signal is increasingly prone to influence by various sources of external noise as trial sizes expand in later stages of development. Notably fewer industry-sponsored CIAS trials are being conducted now than in the early wake of the MATRICS initiative, with a growing tendency to view these trials as inordinately difficult and unlikely to achieve success. In this context, we believe the time is right to take stock and reflect on the challenges identified and lessons learned from industry experience.

## Challenges and Complexities in Multi-center CIAS Trials

The translation of MATRICS guidelines into operational practice has revealed substantial complexities and challenges.

### Practical Issues Associated With the MCCB Include:

- The complexity of this traditional 10-subtest paper-and-pencil-based cognitive assessment battery necessitates substantial training at start-up for raters meeting educational/experience requirements (and for replacement raters during a trial), as well as substantial on-going efforts to maintain rater fidelity during lengthy trials. Training on these traditional tests is particularly time consuming for sites or regions with relatively little cognitive assessment experience.
- The lengthy administration time of approximately 90 min, which contributes to long study visits. Requirements for concurrent assessments, including a co-primary measure, general and specific symptoms (total, negative, and depressive), extrapyramidal symptoms, and labs are considerable; a baseline assessment can span 2 consecutive days. This challenge could not have been detected in the MATRICS normative studies where only the MCCB (and 3 supplemental tests) were administered.
- Some patients and raters experience fatigue, disengagement, and frustration due to the length and complexity of the battery; these burdens can impact data quality and adherence/drop-out rates.^16^
- There are challenges in standardized scoring of paper-and-pencil assessments, leading to scoring errors and lags in identifying invalid/missing data.
- There are challenges intrinsic to handling paper materials and documents, such as requiring raters to remember to select correct alternate forms and then scan or fax paper documents in a timely manner for centralized review and scoring confirmation.
- Cultural adaptation and translation is required and in some cases is impossible (eg, 1 MCCB subtest cannot be administered in certain Asian languages), lengthening study start-up timelines and limiting the global applicability of assessments.
- There is a low correlation between the social cognitive task and the other 9 nonsocial MCCB subtests, compared to the intercorrelations of other subtests^17^; this subtest adds time and is susceptible to cultural differences.^18^
- Costly videotaping is required to conduct comprehensive rater and data quality monitoring during a trial.
- Site- and region-based differences in performance are not uncommon^19–21^ and would be expected on the basis of differences in the many factors (eg, educational standards and quality) that can impact cognitive performance. Such differences highlight the need for continuous real-time data monitoring and quality assurance procedures, which are inefficient with paper-based data.

Since the MCCB was created, non-clinical and clinical research has highlighted an array of extraneous factors that impact performance on cognitive assessments, including diurnal rhythmicity of cognition/arousal, sleep disturbances, exposure to stress, and substance use,^22–24^ all of which complicate CIAS trials (eg, scheduling requirements for consistency in time of day) and contribute noise to cognitive data collection.

### Co-primary Requirement

The co-primary requirement was carried forward by regulators from previous studies attempting to treat cognition and functioning in dementia. However, the MATRICS group did not view any existing measure as sufficiently reliable, valid, and sensitive to recommend a “gold standard” to provide evidence of clinical meaningfulness for CIAS.^25^ There are major challenges associated with the 2 general types of measures that they did recommend considering:

- *Interview-based measures of cognition*: These assessments require a patient and informant to answer questions about the patient’s cognitive impairments and the degree to which they impaired daily functioning over a specified period. For most people with schizophrenia, self-reports of level of cognitive ability and functioning are notoriously inaccurate.^26,27^ Although high-quality informant ratings can be highly accurate, many patients do not have a willing and available informant who sees them with sufficient frequency to provide meaningful ratings_._^25,28^ The result is that an informant requirement can be a substantial barrier to recruitment and one that varies across regions in multi-national trials. When informants are available, they are often limited by a highly selective observational window into the patient’s behavior and can be prone to biases, including systematic cultural differences. There is also the possibility that there is a reactive effect of nominating an individual as an informant, in that their increased attention to the participant during the trial may lead to them discovering other elements of impairment that they had not previously observed. All these factors can increase variability and noise in the data.
- *Performance-based measures of functional capacity*: These tests comprise simulations of real-word activities such as shopping and making other purchases, taking public transportation, or maintaining a conversation.^28–30^ While such measures do not rely on patient or informant reports and demonstrate a closer association with cognitive test data than the interview-based measures,^25,31^ previously developed paper-and-pencil versions have limitations for repeated use in multi-site clinical trials. These limitations can include requirements for a large set of props, lack of alternative forms, extensive off-line scoring and data management requirements, poor psychometrics (eg, ceiling effects, large practice effects), absence of established norms, outdated item content, and vulnerability to cultural and practical variations in how such activities are performed across countries.^32–35^

### 6-Month Trial Duration

The consensus guidelines recommend a 6-month trial duration. This makes sense for studies in dementia, where prevention or slowing of decline, which may require at least 6 months for detection, is a favorable outcome in addition to improvement from baseline. The applicability of a deterioration model to CIAS is debatable. In addition, many cognitive enhancing agents have meaningful effects in much shorter time frames. At the time of the enactment of this requirement, a leading candidate medication was a cholinergic alpha-7 agonist, which manifested potential for loss of efficacy because of tachyphylaxis.^36^ Tachyphylaxis is not an issue for many drugs, however, so requiring all drugs to be tested over an extended duration because some drugs may manifest loss of efficacy is a very broad requirement.

### Summary of Learnings From CIAS Trials

Through 20 years of industry experience, we have learned that the measures and methods prescribed by the MATRICS guidelines are quite challenging to implement in real-world, multi-site, and multi-national trials due to the associated practical complexities, patient/site burden, and trial duration. These factors present substantial barriers to participant enrollment, adherence, and retention, and the scientific merit of some of the guidelines is debatable. In combination, these factors make trials slow to recruit for and expensive to implement, frequently resulting in pressure on sponsors to “speed-up” trials by relaxing standards (eg, by adding sites or countries with limited clinical trial experience, lowering requirements for qualified raters), which can lead to increased variability in scores and compromised data quality.^37^ Furthermore, there is a growing perception among industry decision-makers that the challenges of implementing CIAS trials are so extraordinary that they render allocating further resources to drug development in this area unjustifiable. In this context, we believe the time is right to critically reconsider some aspects of the guidelines that may hinder efforts to bring beneficial new treatments to patients.

## Issues for Reconsideration

Drawing from industry experience in conducting multi-site, multi-national trials, and related scientific developments, we believe several issues relating to the MATRICS guidelines may warrant careful reconsideration based on scientific developments since 2008. We focus here on 6 issues.

### Is It Necessity to Assess All 7 MATRICS Cognitive Domains?

The primary outcome variable in nearly all CIAS trials is a global cognitive composite score, as recommended by the developers of the MCCB. Although the MATRICS guidelines allow investigators to focus on a single cognitive domain, hardly any later phase trials (eg, Biogen’s phase 2 study of BIIB104) have used this strategy. It is questionable whether assessing all 7 MATRICS cognitive domains is necessary to generate a valid or equivalent composite score. Decades of schizophrenia research show that most of the variance in nearly any neuropsychological battery is accounted for by a single dominant general cognitive impairment factor^38–42^; this is true for the MCCB, as well as much shorter and much longer batteries. Furthermore, composite score indices are consistently more strongly associated with level of functional impairment, measured with either everyday functioning or functional capacity, than any single cognitive domain or test.^43–45^

Ten cognitive tests are not required to rigorously index this general factor and some tests appear to contribute little to no variance to the derived composite measure. Most of the variance among the 10 MCCB subtests can be captured by a small subset of domains or tests^41^; a combination of 3 MCCB subtests administered in less than 10 mins correlates strongly with the full MCCB composite score (*r* = .76) while showing comparable psychometrics and validity.^42^ In a study of an extensive battery,^38^ 3 tests that were identical to or substantially equivalent to MCCB tests (Digit Symbol, Hopkins Verbal Learning Test Total Recall, and Letter-Number Sequencing), accounted for 80% of the variance in the composite score for a 9-test battery. Critically, those 3 tests took approximately 13 mins to complete, compared to 88 min (comparable to the MCCB) for the entire battery. Some longer tests currently in the MCCB, such as the Identical Pairs-Continuous Performance Test (which itself takes 13 min to complete), have been reported to contribute 2% of the variance to total scores. Alternative batteries, such as the widely used Brief Assessment of Cognition, which assesses 4 MATRICS domains with 6 subtests in approximately 30 min, generates a composite score that correlates .74 with the full MCCB and demonstrates similar psychometrics and validity.^46–48^ Thus, a shorter battery could effectively index general cognitive impairment and decrease participant and site burden with equivalent scientific rigor compared to the MCCB.

An argument for using a more extensive battery could be to detect specific influences of medications, positive or negative, on specific domains of cognitive functioning. However, the MCCB developers argued that the goal of detection of specific beneficial effects of a treatment would require adding supplemental tests to the core MCCB (ie, composite).^49^ Thus, supplemental measures would be required to demonstrate specific benefits. The implication is that differences across MCCB subtests arising from treatment with a specific agent would not be sufficient to demonstrate differential efficacy (or worsening), which undermines the argument for full coverage of all 7 cognitive domains.

### Could Alternative Computerized Cognitive Batteries Be Useful?

The MATRICS guidelines indicate that a battery “equivalent” to the MCCB would be acceptable, but no definitional criteria for equivalence were provided. There are many apparent advantages of supervised computer-assisted batteries (eg,^48,50,51^) for large-scale trials, including much lower tester training demands, semi-automated presentation of instructions and tasks to facilitate standardized administration, automated scoring to reduce scoring errors (and data entry costs), automated audio recording for data quality monitoring, simpler cloud-based data storage, efficient data management, and real-time data analytics. These advantages are sometimes offset by disadvantages. For eg, in contrast to most opinions, computerized assessments sometimes have more missing data than paper and pencil assessments.^38^ Reasons for this are not completely certain but may include participant challenges in technology-based interactions or relaxed supervision by testers during the assessment process. In any case, the uncertainty about what constitutes an equivalent battery is a risk that disincentivizes sponsors from creatively considering alternatives and there have been public debates about this issue.^52–54^ Furthermore, there is a perception among industry drug developers that regulatory requests to collect additional supporting validation data for alternative batteries would likely be very extensive and challenging to accomplish. Clearly defined guidance for equivalence, particularly if the guidance is based on existing data, could encourage innovative assessment approaches.

### The Co-primary Outcome Requirement May Not Be Realistic or Practical

The co-primary requirement for CIAS trials originated in an entirely different treatment model. The requirement originated in Alzheimer’s Disease (AD) studies, where it is known that reduced ability to perform functional skills arises from cognitive impairments associated with Mild to Moderate AD. Thus, co-primary outcomes were conceptualized as detecting *regaining* elements of prior functioning that had definitively been present and had deteriorated with dementia. Regaining skills in concert with cognitive enhancement could be substantiated by recovery of the ability to perform previous functional skills, indexed by observer observation. Furthermore, as some skills that deteriorated because of early effects of dementia might be restricted by relatives because of concerns regarding risk (eg, financial management, driving), performance-based simulations focused on these specific and commonly performed skills could also be used.^55,56^

To logically apply the same model to schizophrenia studies, however, evidence of prior adequate functioning and subsequent loss would be required, which may pose an insurmountable challenge in a clinical trial. Common functional capacity measures such as the USCD Performance-Based Skills Assessment include skills such as financial activities, map reading, managing transit schedules, and meal preparation. Even at this basic skill level, we know that many people with schizophrenia never fully acquired the skills assessed by co-primary measures (see^57^). Thus, a requirement for improvement on a co-primary may pose an extraordinary burden, in that participants may need to manifest improvement in the performance of functional activities that they never adequately performed before.

The notion that cognitive improvements alone may not induce performance of activities that were never previously performed is not purely speculative. Previous successful studies of cognitive training in healthy older people found substantial gains in cognition and improved performance on previously acquired functional skills, but novel ADL activities were not acquired regardless of the level of cognitive gains.^57,58^ Similarly, in schizophrenia, computerized cognitive training (CCT) administered alone typically results in substantial gains in cognition but not in functional capacity or real-world functioning; in contrast, CCT with concurrent psychosocial interventions do typically lead to much more substantial gains in both cognition and functioning.^58,59^ Thus, the conclusion that because a pharmacological treatment offered without psychosocial intervention or support fails to induce acquisition of novel functional skills means that the treatment does not meaningfully enhance cognition seems inconsistent with the evidence regarding skills learning accrued since the initial MATRICS guidance. It may be time to consider alternative approaches to assessing the functional meaning of treatment-related improvements in cognitive task performance.

### Trial Design Guidelines for Monotherapy Agents

CIAS trials over the past 20 years have evaluated adjunctive therapies and the MATRICS guidelines clearly mandate a placebo-controlled design for add-on trials. Guidelines for monotherapy trials are much less extensively developed. However, mechanistically novel monotherapies that address multiple symptom domains, possibly including cognitive impairment, are now emerging, including a new generation of cholinergic muscarinic receptor agonists.^60,61^ For eg, across phase 2 and 3 trials in acute schizophrenia, monotherapy with KarXT, an M1/M4 receptor-preferring agonist, has shown replicable improvements on Positive and Negative Syndrome Scale (PANSS) total, positive, and negative subscales, as well as improvements in cognition among patients with baseline cognitive impairment.^62,63^ Similarly, monotherapy with Cariprazine, a mechanistically novel dopamine D3-prefering D3/D2 receptor partial agonist and 5-HT1A partial agonist, has been reported to have benefits across positive, negative, and cognitive domains.^64,65^ Although the MATRICS guidance recommends that cognitive benefits of monotherapy agents should also be demonstrated using a placebo-controlled design with stable patients, complications with this approach are explicitly acknowledged.^12,13^ Thus, there are use cases that did not exist at the time of the MATRICS initiative that require regulatory consideration.

Historically, the FDA’s position has been that correlational analyses are not adequate to substantiate a lack of relationship between change scores for clinical symptoms and cognitive impairments. However, if 2 independently measured variables are found to be uncorrelated at baseline (as is common for psychosis and cognition) and changes in the 2 variables are also uncorrelated in adequately powered studies, it is not at all clear how the variables could be argued to be related or, even more challengingly, overlapping. Notably, in the studies of KarXT monotherapy, the drug separated from placebo on 2 outcomes (cognitive performance and clinical symptoms), with the baseline and change scores for the 2 outcome variables found to be uncorrelated. Thus, an argument that changes in psychosis caused better performance on a cognitive assessment is hard to defend.

The FDA has previously provided a limited endorsement of cognitive benefits in a clinically effective antidepressant (vortioxetine) based on 2 separate trials conducted in adult patients with major depression. In 1 3-arm trial in which participants were randomized to placebo, 10 mg, 20 mg^66^, both doses of vortioxetine were superior to placebo for depression as well as for the DSST. In another 2-arm acute treatment study,^67^ both duloxetine and vortioxetine were superior to placebo for antidepressant efficacy. Vortioxetine, but not duloxetine, separated from placebo on the DSST, suggesting across studies that a treatment had 2 benefits compared to placebo rather than only 1.

Following the logic of the vortioxetine studies, 1 solution would be a 3-arm acute treatment study, with the putatively broad-spectrum agent, an existing antipsychotic medication without evidence of cognitive benefit in previous studies, and a placebo control. However, this design would require exposure to placebo of substantial samples of clinically stable people with schizophrenia. The pivotal vortioxetine study was an acute treatment study and KarXT has shown superiority for both cognition and PANSS Total Scores compared to placebo in acute treatment studies. The length of the pivotal vortioxetine study was 8 weeks and the KarXT study was 5, shorter than the 6-month requirement currently in place for CIAS. Current regulatory logic suggests that the lack of correlation between 2 change scores fails to refute a lack of influence of 1 process on the other. In that context, it might be challenging to convince regulators that equivalent, historical superiority of 2 treatments for symptoms over placebo and current superiority of 1 treatment to the other for cognition means that there is a difference in terms of cognition.

If, as described above, a monotherapy treatment demonstrates short-term clinical efficacy, including cognitive benefit, and acceptable safety/tolerability across placebo-controlled phase 2 and 3 trials with acute patients, the value of placebo-controlled trials with stable patients is uncertain, as are the conceptual and ethical considerations associated with requiring placebo in stable patients. A placebo-controlled monotherapy study of stable patients would require a washout from prior stability-inducing treatments. There is a greater than 2-fold increase in risk of relapse among placebo-treated patients (35%) with schizophrenia compared to antipsychotic-treated patients (14%) over 3 months^68^; symptom relapses or even associated symptom increases/fluctuations in a placebo group would add noise and jeopardize cognitive data quality. Furthermore, the high relapse risk will likely contribute to reluctance in treatment providers, family members, and patients to participate given the major psychosocial disruption and potential neurotoxic impact associated with relapse.^69,70^

An alternative to these clinical, ethical, and scientific risks would be a superiority design with an active comparator drug known to be cognitively neutral based on previous large-scale evidence, such as the Clinical Antipsychotic Trials of Intervention Effecticeness (CATIE) trial, wherein several different medications were found to have minimal cognitive benefits or liabilities (eg, olanzapine, quetiapine, and risperidone). There are several variants of a “sustained stability combined with superiority” design that could be adopted, including a blinded randomized “stay vs switch” of clinically stable patients proven to manifest cognitive impairments while currently treated with a single treatment, to either stay on current treatment or switch to the dual-benefit monotherapy agent. The advantage of this design is that all patients are stable in their prior treatment, so 50% of patients would not be at risk to develop clinical instability on a new agent, reducing attrition risk. The weakness of this design is that there is only a single-drug comparison, which could lead to disagreements about labeling the benefit as superior to only one of the many currently available antipsychotic medications. It is important to understand that large studies (CATIE) and meta-analyses suggest that the most used medications (and the ones which are the “base medication” in previous augmentation studies targeting cognition) are equivalent in their lack effects on cognition. Requiring that the already understood lack of cognitive benefit be proven repeatedly with separate trials for each possible antipsychotic comparator would cripple drug development with this strategy.

The other alternative is to recruit clinically stable patients proven to manifest current cognitive impairments on a variety of approved antipsychotic treatments, who are then randomized to either the dual-benefit monotherapy agent or another treatment (eg, risperidone). The advantage of this design is that post-treatment cognitive performance, associated with the comparator vs the dual-benefit monotherapy agent could be compared to pre-randomization cognitive performance across an array of different baseline treatments. The disadvantage is that some patients could destabilize on either the comparator or the dual-benefit monotherapy agent and such patients could not be examined for “stability and superiority” but could provide information about potential cognitive gains even in the presence of new-onset clinical instability. Thus, with this design, all patients are theoretically at risk for destabilization because their medications are changed. However, such a study would collect evidence of improvement in cognitive function in patients who remain clinically stable as well as those who destabilize. It is worth noting that the European Medicines Agency guidelines suggest that the comparison of new agents with older agent for treatment of the same conditions does not require a placebo-controlled design.

### Enrichment Strategies

In line with MATRICS guidelines, no industry-sponsored CIAS trials to date have prospectively enriched samples by selecting participants based on severity or stability of cognitive impairment. The rationale for the MATRICS guideline for enrolling all comers was based on the idea that the vast majority of people with schizophrenia show some degree of under-performance compared to parental education-based expectations and the findings of a generally normal distribution of scores in schizophrenia.^71–73^ However, enrollment criteria based on severity of clinical symptoms (eg, on the PANSS or Montgomery-Asberg Depression Rating Scale) are uniformly implemented in regulatory trials to enrich samples for the clinical phenomenon of interest. There is no requirement that non-depressed participants should be enrolled in antidepressant efficacy studies, regardless of any scientific or regulatory interest in the effects of antidepressants in healthy controls, as it would be seen as unethical to expose them to the risks. Since approximately 20% of people with schizophrenia do not show clinically meaningful cognitive impairment (ie, performing within 1 standard deviation of healthy normal)^2^ based on current test performance, the same logic could apply to cognition as clinical symptoms, namely that individuals who have meaningful current symptoms are the target population for whom pro-cognitive drugs are developed. The argument that everyone with schizophrenia is performing worse than expectations is based on utilization of detailed information about familial levels of educational and cognitive performance that would be impossible to collect in valid manner a multi-site clinical trial.

Cluster analytic studies consistently identify a subgroup of patients described as “neuropsychologically normal,” who differ from the majority of people with schizophrenia in terms of premorbid cognitive function, clinical course, genetic, and structural/functional neuroimaging characteristics.^74^ Furthermore, current cognitive functioning is not correlated with the severity of functional disability among unimpaired patients.^75^ Within this higher-functioning subgroup, there can also be concerns about ceiling effects, which are compounded by the documented screening to baseline intraindividual variability on the MCCB,^76^ as well as differential “discriminating power” or sensitivity of normed tests across the range of abilities. As known for years,^77^ optimal discrimination between 2 groups of different abilities and detection of change within individuals is best achieved by testing with similarly reliable items that reflect the full range of task difficulty, in approximate proportion to the overall normal distribution of scores (ie few very hard, few very easy, and the majority in the mid-range with graduated difficulty). For higher functioning participants, the same tests (eg symbol coding) are less difficult, less variable, and thus less able to detect within-person change. Although some drugs can enhance cognition among those within or even above the normal range, particularly stimulant medications,^78^ most drugs approved for conditions with impaired cognition have been found not to enhance cognitive performance ie in the average range (eg,^79^). Several studies have found modest and highly delineated benefits of cholinergic or glutamatergic agents in individuals not selected for cognitive impairment, with no evidence of improvements in overall composite cognitive scores.^80–82^

Aside from severity, it may also be useful to enrich for stability of cognitive performance. Clinical trial samples targeting clinical symptoms (eg, depression, positive symptoms) are commonly enriched for symptom stability between screening and baseline assessments. The large empirical database of CIAS clinical trials amassed over the past 2 decades could be used to define empirically based cutoff scores for extraordinary worsening or improvement (while accounting for typical practice effects^76^) from screening to baseline, with such changes likely reflecting influences of external factors such as variable engagement/effort, fatigue, or substance use.

The majority of patients enrolled in CIAS trials manifest stable performance and predictable practice effects from screening to baseline, demonstrated by retest analyses of participants randomized to placebo.^76^ However, an analysis of a large MCCB data set from CIAS trials^76^ found that 11% of the participants worsened by 0.5 SD or more from screening to baseline and 25% improved by 0.5 SD or more from screening to baseline assessment. There was a divergent course during reassessment during the trial, wherein patients (11%) who worsened by 0.5 SD or more from screening to baseline demonstrated larger placebo effects than those whose retest change scores were less than +/− 0.5 SD, who in turn had still larger placebo effects than those who had relatively large (> 0.5 SD) score increases from screening to baseline. Thus, placebo-related changes in performance from screening to baseline were marked by changes in test performance in opposite directions over time. Similarly, post-hoc analyses of the phase 3 encenicline trials found that subjects who showed atypically large screening to baseline changes had a major adverse effect on the overall results.^37^

Treatment adherence has been a major challenge in some CIAS trials^37,83^ and enriching for potential treatment adherence may be another reasonable approach to enhance signal detection. This may be accomplished by examining adherence to simple assessment procedures, such as completing brief ecological momentary assessment (EMA) surveys during run-in. Adherence in the first day to first week in EMA studies has been found to robustly correlate with adherence, to both EMA and other assessments, over the course of study protocols.^84^

### Trial Duration

The 6-month minimum trial duration in the MATRICS guidelines appears be a historical artifact and not linked to recent evidence regarding data on the time course of drug-related cognitive benefits. The factors that may have that influenced its selection are described below:

(1). The dual targets of improvement of cognitive performance and reducing longitudinal cognitive decline in pharmacological treatment studies for AD and related dementias, which requires a 6-month or more follow-up to be detected with validity.

(2). A leading drug candidate for schizophrenia in the 2000’s was vulnerable to tachyphylaxis, requiring a systematic assessment of reductions in efficacy over time.

In contrast to the rationale for these requirements, some cognitive enhancers (eg, simulants, caffeine, nicotine) show meaningful effects within a matter of hours or days, raising the question of whether a 6-month trial is always required and whether sustained efficacy should be required to be demonstrated in an augmentation trial. Notably, pivotal augmentation trials for depressed patients with MDD who had inadequate response to current antidepressant treatment have led to approvals with 6 to 8-week trial designs for several different medications, including aripiprazole,^85^ brexpiprazaole,^86^ and cariprazine.^87^ A meta-analysis for pharmacological treatment of acute bipolar depression, commonly with augmentation therapies, reported on 7528 participants who were randomized to drug while 3,920 were assigned to placebo.^88^ The median duration of acute treatment was 8 weeks (interquartile range = 6–8), suggesting much shorter trials are the norm for augmentation therapy studies in other indications.

In real-world clinical practice, we suspect that many clinicians (and patients) would not wait 6 months to decide to continue with any new treatment. Clinicians who treat people with schizophrenia are used to making treatment change decisions on the basis of lack of antipsychotic response, which can be identified as early as 3 days^89^ and confirmed at 2 weeks.^90^ Instead of uniformly requiring a 6-month trial, rationally linking trial duration to the speed of the expected cognitive effect, based on a drug’s mechanism of action, may be appropriate. Shorter trials, as appropriate, would directly decrease patient and site burden.

## Some New Approaches to Consider

Our knowledge base regarding the complexities and obstacles to collecting high-quality cognitive data in large-scale clinical trials has grown since the MATRICS guidelines were established. Implementation of these guidelines in large-scale CIAS trials has proven operationally and practically complex. The high burden for patients, sites, and data monitors may translate into key sources of error variance in the cognitive data that are collected, undermining efforts to identify a treatment signal. In addition, new findings related to composite cognitive scores, functional capacity assessments, and the time course of effects of potential pro-cognitive agents raise questions about the current scientific rationale for some elements of the original guidelines.

To help our field move toward a more streamlined and efficient CIAS drug development pathway we offer the following refinements for consideration.

- Allow shorter cognitive batteries that do not require coverage of all 7 MATRICS domains but yield meaningful and validated composite scores.
- Establish clear, practically achievable criteria for suitable alternative cognitive batteries to address training, standardization, and data monitoring and quality control challenges.
- Eliminate the concurrent co-primary requirement to demonstrate the functional significance of drug-related cognitive benefits. Assessment of ultimate functional impact could be separated from initial evaluation of efficacy for cognitive enhancers in a 2-tiered approval process, such as is used with antipsychotic treatment. For eg, functional effects could be evaluated in longer-term efficacy/safety follow-up studies (eg, post-marketing requirements) using standard functional outcome assessment strategies, including rating scales or milestone achievements (eg, transitioning to independent living or beginning work/school), particularly in enriched populations receiving specialized interventions such as supported employment.^91^ Several innovative, and potentially more sensitive, digital technology-based assessment approaches may also be useful for demonstrating functional benefits while minimizing participant/site burden. These include remote active digital phenotyping (eg, EMA assessments of functioning, functional capacity, and cognition during the course of daily life) and passive sensor-based digital phenotyping (eg, actigraphy, Global Positioning System data effortlessly collected from a smartphone or wearable device) metrics of elemental aspects of real-world daily functioning (eg, engaging in productive and/or social activities, frequency of leaving one’s home, daily activity level, daytime napping).^92,93^ Remote assessment approaches have been shown to be feasible in studies as long as a year and could detect many predefined functional changes if they were to occur.^94–96^
- Support enrichment strategies that focus clinical trials on the target population most likely to benefit from cognitive enhancing treatments, namely, those with temporally stable, clinically meaningful cognitive impairment.
- Develop rational guidelines for monotherapy trials considering the scientific and practical risks associated with lengthy outpatient placebo-controlled trials. These guidelines will need to consider the best comparator strategies, including randomized switching strategies for the new target agents.
- Decrease the minimal trial length requirement and allow for duration flexibility based on a drug’s mechanism of action and associated time course for producing effects on cognition. Durability of efficacy for cognition could be examined with the long-term real-world evidence strategies described above, which could also consider long-term adherence and late-occurring adverse events.

## Conclusions

Refinements to current MATRICS-based CIAS trial methods and regulatory requirements based on learnings arising since MATRICS guidance in 2008 may help us advance towards more efficient and effective drug development, sustain industry investment in CIAS trials, and drive innovation into finding new ways to help people living with schizophrenia achieve their functional goals. Importantly, these learnings directly extend to drug development efforts for other neuropsychiatric disorders characterized by cognitive impairment. For eg, although “CIAS” is distinguished on a regulatory basis from cognitive impairment associated with bipolar disorder or major depression, similarities in the qualitative profiles of cognitive impairment across these disorders far outweigh differences.^3–7^ Although the relative severity of impairment on cognitive tests in bipolar disorder and depression commonly differs from CIAS, the persistence (even across euthymic states), impact on functional outcomes, and neural/genetic correlates do not^40,97–103^ and much of the severity difference could be attributable to the influence of differences in premorbid functioning, seen at the time of the first episode.^94^ With evidence-based, achievable clinical trial guidelines, similar pro-cognitive pharmacological interventions have the potential to benefit patients across these traditional nosological boundaries. We hope the perspective offered in this article contributes to an open exchange among all stakeholders about possible enhancements to drug development methodology for treatment of cognitive impairment in schizophrenia and other neuropsychiatric conditions.
